# Leucine-rich diet induces a shift in tumour metabolism from glycolytic towards oxidative phosphorylation, reducing glucose consumption and metastasis in Walker-256 tumour-bearing rats

**DOI:** 10.1038/s41598-019-52112-w

**Published:** 2019-10-29

**Authors:** Laís Rosa Viana, Natália Tobar, Estela Natacha Brandt Busanello, Ana Carolina Marques, Andre Gustavo de Oliveira, Tanes I. Lima, Gabrielly Machado, Bianca Gazieri Castelucci, Celso Dario Ramos, Sérgio Q Brunetto, Leonardo Reis Silveira, Anibal Eugenio Vercesi, Sílvio Roberto Consonni, Maria Cristina Cintra Gomes-Marcondes

**Affiliations:** 1 Laboratory of Nutrition and Cancer, Department of Structural and Functional Biology, Biology Institute, University of Campinas, Campinas, SP Brazil; 2 Division of Nuclear Medicine, Department of Radiology, School of Medical Sciences, University of Campinas, Campinas, SP, Brazil; 3 School of Medical Sciences, Department of Clinical Pathology, State University of Campinas, Campinas, SP Brazil; 4 Obesity and Comorbidities Research Centre, Biology Institute, University of Campinas, Campinas, SP, Brazil; 5 Laboratory of Cytochemistry and Immunocytochemistry, Department of Biochemistry and Tissue Biology. Biology Institute, University of Campinas, Campinas, SP Brazil

**Keywords:** Cancer metabolism, Endocrine cancer, Cell biology, Endocrine system and metabolic diseases

## Abstract

Leucine can stimulate protein synthesis in skeletal muscle, and recent studies have shown an increase in leucine-related mitochondrial biogenesis and oxidative phosphorylation capacity in muscle cells. However, leucine-related effects in tumour tissues are still poorly understood. Thus, we described the effects of leucine in both *in vivo* and *in vitro* models of a Walker-256 tumour. Tumour-bearing Wistar rats were randomly distributed into a control group (W; normoprotein diet) and leucine group (LW; leucine-rich diet [normoprotein + 3% leucine]). After 20 days of tumour evolution, the animals underwent ^18^-fludeoxyglucose positron emission computed tomography (^18^F-FDG PET-CT) imaging, and after euthanasia, fresh tumour biopsy samples were taken for oxygen consumption rate measurements (Oroboros Oxygraph), electron microscopy analysis and RNA and protein extraction. Our main results from the LW group showed no tumour size change, lower tumour glucose (^18^F-FDG) uptake, and reduced metastatic sites. Furthermore, leucine stimulated a shift in tumour metabolism from glycolytic towards oxidative phosphorylation, higher mRNA and protein expression of oxidative phosphorylation components, and enhanced mitochondrial density/area even though the leucine-treated tumour had a higher number of apoptotic nuclei with increased oxidative stress. In summary, a leucine-rich diet directed Walker-256 tumour metabolism to a less glycolytic phenotype profile in which these metabolic alterations were associated with a decrease in tumour aggressiveness and reduction in the number of metastatic sites in rats fed a diet supplemented with this branched-chain amino acid.

## Introduction

Cachexia affects up to 80% of advanced cancer patients and is responsible for almost 30% of all cancer-related deaths^1^. Cachexia related to cancer, termed cancer cachexia, is a multifactorial syndrome that leads to an involuntary weight loss associated with poor quality of life, poor prognosis, and high mortality. The main cause of weight loss is skeletal muscle wasting^2^ that leads to muscle activity impairment. Thus, in order to prevent or to treat skeletal muscle wasting, some nutritional strategies have been used. In this regard, leucine supplementation has mainly been investigated due to its potential to increase protein synthesis in skeletal muscle. Alone or together with other branched-chain amino acids (BCAAs), including valine and isoleucine, leucine has stimulatory actions on skeletal muscle protein synthesis by acting as a cell signalling molecule leading to the activation of the mechanistic target of rapamycin (mTOR) pathway, the key cellular protein synthesis pathway^3,4^. Moreover, leucine can also act as an energetic molecule. Once in the cell, this amino acid is converted into α-ketoisocaproate by BCAA aminotransferase (BCAT) after which α-ketoisocaproate is converted to isovaleryl-CoA by BCAA α-ketoacid dehydrogenase (BCKD) and finally used in the citric acid cycle (TCA) to produce ATP^5^. Furthermore, leucine increases the peroxisome proliferator-activated receptor gamma coactivator-1 alpha (PGC-1α) expression leading to mitochondrial biogenesis and improving oxidative metabolism in skeletal muscle^6–8^.

Although the effects of leucine are well-described in skeletal muscle, the effects of leucine on tumour tissues are still unknown, specifically with respect to the mechanism of tumour growth. Therefore, considering the potential benefits of leucine treatment of cancer-associated muscle wasting, we aimed to describe the effects of a leucine-rich diet in the cancer cachexia Walker-256 tumour model.

## Results

### Leucine-rich diet decreases Walker-256 tumour glucose analogue (^18^F-FDG) uptake and metastases formation

**Figure 1:**
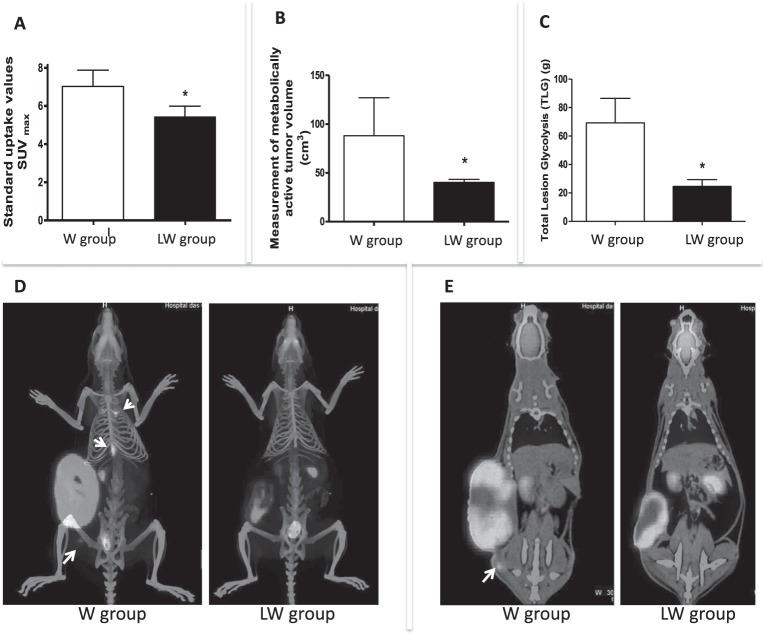
Leucine-rich diet decreased tumour ^18^F-fludeoxyglucose (FDG) uptake and reduced number of metastases sites. (**A**) Graphic showing the standardized uptake values (SUV_max_) values; (**B)** Metabolic tumour volume (MTV; cm^3^); (**C**) – Total lesion glycolysis (TLG, SUV [mean] × metabolic tumour volume) in Walker (W/Control) and leucine (LW) tumour-bearing groups; (**D**) Computed tomography (CT) image of hard tissues analysis; and (**E)** CT image of soft tissues analysis. The head arrows indicated metastases site. N = 4 animals per group for this analysis procedure. Graphics represent mean ± standard deviation. For details, see the Methods section. *P < 0.05 significance compared to W group (Student’s *t*-test).

**Figure 2:**
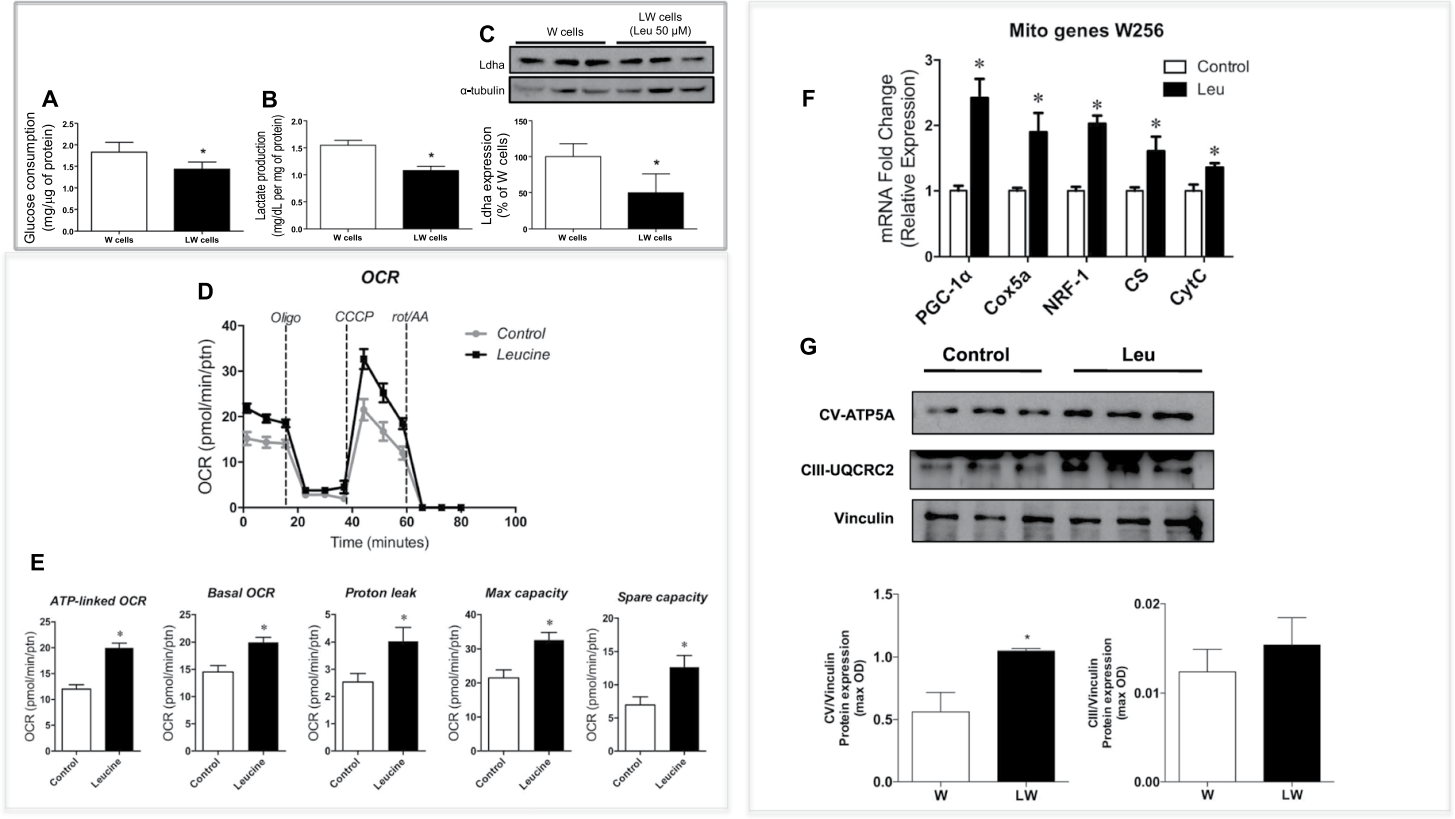
*In-vitro* assays: Leucine reduced glucose consumption and lactate production and increased oxygen consumption by Walker-256 tumour cells. (**A**) Glucose consumption (mg/µg protein); (**B**) Lactate (Ldha) production (mg/dL per mg protein); (**C**) Ldha expression (% of W cells; representative image for western blot experiments, and graphics showing quantitation of western blot image; the blots images were cropped; full-length blots are included in Supplementary Figure) in Walker-256 cultured cells treated or not with 50 µM L-leucine for 24 h; (**D**) Seahorse traces. Oligomycin (1 µM) was used to inhibit ATP synthase, protonophore carbonyl cyanide m-chlorophenyl hydrazone (CCCP) (2 µM) to uncouple mitochondrial OXPHOS, and rotenone (rot)/antimycin (AA) (1 µM) to block mitochondrial respiration and determine non-mitochondrial oxygen consumption rate (OCR); (**E**) Bar graphs of the calculated ATP-linked OCR (calculated by subtracting the uncoupled [after the addition of oligomycin] from the basal OCR), basal OCR, proton leak, and maximal and spare capacities (determined by subtracting basal from the CCCP-induced OCR). Non-mitochondrial OCR values were subtracted from all data before being used for the analyses. All Seahorse measurements were normalized by sample protein content (Bradford assay); (**F)** Correspond to the PCR analyses from tumour cells showing the expression of mitochondrial enzymes such as PGC1a, COX5a, NRF-1, CS, and Cytc; (**G**) The tumour cells were lysed and analysed by immunoblotting with antibodies against mitochondrial respiratory complexes ATP5A, and UQCRC2, showing the Western blot images and bar graphs analyses (the blots images were cropped; full-length blots are included in Supplementary Figure). *P < 0.05 compared to control cells (Student *t*-test).

**Figure 3:**
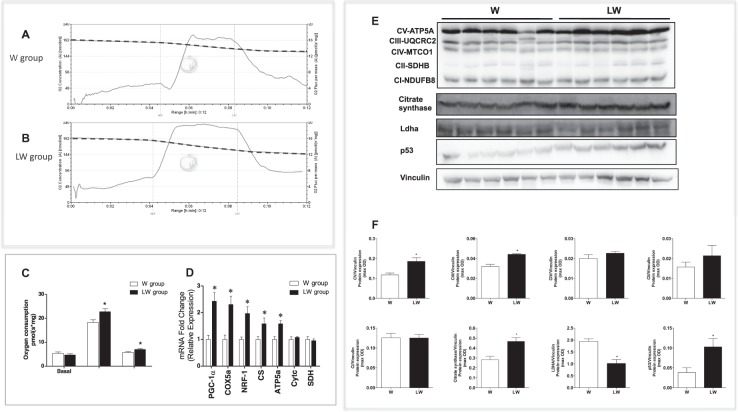
*In-vivo* assays: Leucine rich-diet increased oxygen consumption by Walker-256 tumour biopsies and both mitchocondiral genes and proteins expression. (**A**,**B**) Representative traces of tumour respiration in the W and LW groups, respectively, in which O_2_ concentration (dashed line) is expressed as nmol O_2_/mL and O_2_ flux per mass (continuous line) is expressed as ρmol O_2_/sec mg tissue; (**C**) Bar graphs show the data of O_2_ consumption (ρmol O_2_ / s. mg tissue) compiled from the respiration traces comparing the tumour biopsies from rats under W or LW group. (**D**) Correspond to the polymerase chain reaction (PCR) analyses from tumour tissue from the W *versus* the LW group showing the expression of mitochondrial enzymes, such as peroxisome proliferator-activated receptor gamma coactivator-1 alpha (PGC1α), cyclooxygenase (COX)5a, nuclear respiratory factor (NRF)-1, citrate synthetase (CS), ATP synthase (ATP)5a, cytochrome C (Cytc), and succinic dehydrogenase (SDH); (**E**) Western blot analysis images from mitochondrial respiratory complexes: V (ATP5A [adenosine triphosphate synthase 5A]), III (UQCRC2 [ubiquinol-cytochrome c redutase 2]), IV (MTCO1 [mitochondrial cytochrome c oxidase 1]), II (SDHB [succinate dehydrogenase complex subunit B]), I (NDUFB8 [NADH dehydrogenase 1β subcomplex subunit 8]), and CS Lhda, and p53 expressions in tumour biopsies (the blots images were cropped; full-length blots are included in Supplementary Figures); (**F**) Bar graphs indicating western blot analysis representing values of maximal optical density (max OD). Vinculin was used as a housekeeping protein. The results are presented as mean ± S.D. *P < 0.05 by Student’s t test. For details, see the Materials and Methods section. Biopsy assay: Respiration was evaluated in a medium MiR05 at 37 °C containing 10 mM glutamate plus 5 mM malate as substrates. ADP (1 mM) and carboxyatractyloside (CAT, 12 μM) were added during the experiments.

Tumour glucose uptake measured using standardised uptake value (SUV)_max_ of ^18^F-fludeoxyglucose (FDG) decreased by 22.6% in the leucine-rich (LW) group (P ≤ 0.05) (Fig. 1A). Analysing the number of metastatic site found in both groups, we observed a lower number in the leucine group (normal diet [W]: 3.33 ± 1.2 *versus* LW: 1.75 ± 0.9; P = 0.0399). We also found a reduction of 54% in metabolic tumour volume (MTV) (total volume minus necrotic tissue volume; P = 0.05) in the LW group (Fig. 1B) and a decrease in total lesion glycolysis (TLG) (64% lower TLG; P = 0.034 [Fig. 1C]), which was measured by the images from computed tomography (CT) scans of bone and soft tissues windows (Fig. 1D,E). Corroborating with these findings, the *in vitro* assay showed that tumour cells exposed to leucine (50 µM for 24 h) consumed less glucose (21%; P = 0.0346) and produced less lactate (30%; P = 0.0012) (Fig. 2A and B, respectively), associated with an increase in total protein (data not shown). In accordance with these data, leucine treatment caused Walker-256 tumour cells to reduce the expression of lactate dehydrogenase ([Ldha] 50% lower; P = 0.0265; Fig. 2C), showing that leucine solely led to changes in the metabolism of these tumour cells as verified in tumour tissues. The decrease in protein expression of Ldha was also observed in the tumour biopsies from the LW group (Fig. 3E,F).

### Leucine induces a shift in Walker-256 tumour metabolism from glycolytic towards the oxidative phosphorylation (OXPHOS) pathway in both *in vivo* and *in vitro* studies

**Figure 4:**
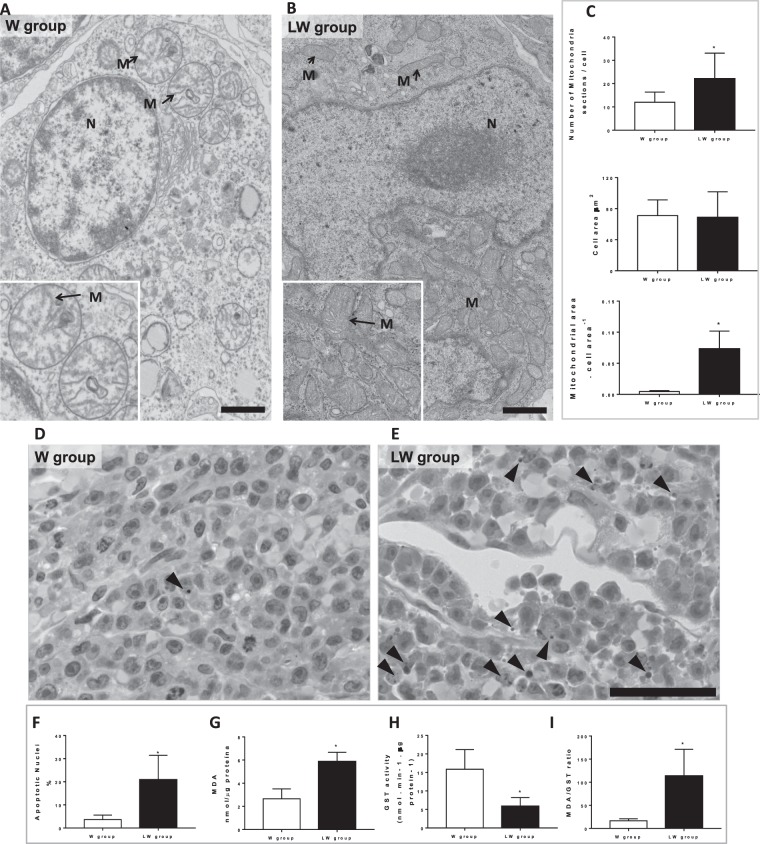
Leucine-rich diet led to mitochondrial biogenesis in Walker-256 tumour tissue. (**A,B**) Representative transmission electron microscopy (TEM) micrographs of Walker-256 tumour from animals fed a control diet (W group) and a leucine-rich diet (LW group); details show the cristae (arrow) of the mitochondria in each group. N = 3 animals per group. N = nuclei; M = mitochondria. Scale bar = 2 μm. (**C**) Graphics of an average number of mitochondrial sections per cell, average cell area (µm^2^), and average mitochondrial area per cell area (analysis accessed from counting from minimal 10 cells per animal from each group). (**D,E**) Representative photomicrographs of W and LW tumour groups. Observe apoptotic nucleus (head arrows). Scale bar = 50 μm. N = 3 animals per each group. (**F**)– Graphs of apoptotic nuclei (%) of W and LW tumour accessed by haematoxylin and eosin staining (analysis counting used 20 different areas from 3 animals per each group). (**G**–**I**) Representative analyses of malondialdehyde (MDA) content, glutathione-S-transferase (GST) activity, and the MDA/GST ratio from tumours of the W and LW groups. N = minimum of eight animals per group. For further details see the Material and Methods section. Values are means ± standard deviation. *P < 0.05 compared to W (Student’s *t*-test).

Oxygen consumption supported by glutamate plus malate was evaluated in fresh tumour biopsies from both groups with the addition of adenosine diphosphate (ADP) and carboxyatractyloside (CAT) during the experiments. Figure 3A,B show typical traces of mitochondrial respiration rates in both experimental groups (W versus LW), in agreement with functional mitochondria. Figure 3C shows that a leucine-rich diet stimulated a significant increase in mitochondrial respiration in both phosphorylating (ADP, 24.3%; P = 0.0053) and resting (CAT, 21.7%, P = 0.0121) states. In line with these mitochondrial respiratory function data, the expression of some mitochondrial genes, including PGC-1α, nuclear respiratory factor (NRF)-1, cyclooxygenase (COX)5a, citrate synthase (CS), and mitochondrial respiratory chain complexes V (ATP5A), were increased in tumour tissues of the LW group (Fig. 3D). In addition, protein expression of ATP5A and III (ubiquinol-cytochrome c reductase 2 [UQCRC2]) and also the expression of CS were significantly increased, while the expression of Ldha was reduced in the tumour biopsies from the LW group (Fig. 3E,F). These molecular results corroborate the increase in mitochondrial volume and density in tumours from the LW group as observed in transmission electron microscopy (TEM) images (Fig. 4A–C). Taken together these data demonstrate that the LW diet caused a metabolic shift from glycolytic to OXPHOS in tumour biopsies.

In order to investigate whether leucine by itself could shift metabolic reliance from glycolysis to OXPHOS as observed in Walker-256 tumour biopsies, we performed an *in vitro* study exposing the isolated Walker-256 cells to leucine (50 µM) for 24 h. Mitochondrial bioenergetics were assessed by measuring oxygen consumption rate using the Seahorse XF24 Analyser. Using the mitochondrial inhibitors oligomycin, rotenone, antimycin, and the protonophore CCCP, we assessed: basal respiration, ATP-linked respiration (portion of respiration associated with ATP production), maximum respiratory and spare capacities, which reflect OXPHOS integrity and electron transport chain capacity. The ATP-linked and basal oxygen consumption rates (OCR), proton leak, maximal respiration, and spare capacity were all significantly increased in leucine-treated Walker-256 cells, suggesting that leucine treatment also induced mitochondrial oxidative capacity *in vitro* (Fig. 2D,E). This was confirmed by the reduction in lactate production and Ldha protein expression in leucine-treated cells (Fig. 2B,C). In agreement, mitochondrial biogenesis was robustly induced as indicated by the increased gene expression of PGC-1α, NRF-1, COX5a, CS, and cytochrome (Cyt)C (Fig. 2F) and also by higher protein expression levels of Complex V (ATP5A) in leucine-treated cells (Fig. 2G). Taken together, these results suggest that leucine per se appears to be capable of inducing metabolic reliance on OXPHOS both *in vivo* and *in vitro*.

### Leucine rich-diet stimulates oxidative stress in Walker-256 tumour biopsies

This shift in tumour metabolism found in tumours of the LW group was accompanied by an increase in mitochondrial area and density (Fig. 4A–C). Interestingly, these mitochondrial alterations stimulated by the leucine diet did not benefit the tumour tissue since no additional growth was observed. This finding may be related to a higher number of apoptotic nuclei (LW was 5.7-fold higher than W group; Fig. 4D–F) and oxidative stress in tumour biopsies from LW group. The increase in oxidative stress was associated with an increase in tumour malondialdehyde (MDA) content (2.2-fold higher in the LW group *versus* the control; Fig. 4G) and lower anti-oxidative response since there was a decrease in activity of glutathione-S-transferase (GST) and a higher MDA/GST ratio (63% reduced in GST, and MDA/GST ratio was 6.9-fold higher in the LW group; Fig. 4H,I, respectively). In agreement with these results, protein expression of the pro-apoptotic p53 was significantly higher in the tumour tissue from the LW group (Fig. 3E,F).

**Figure 5:**
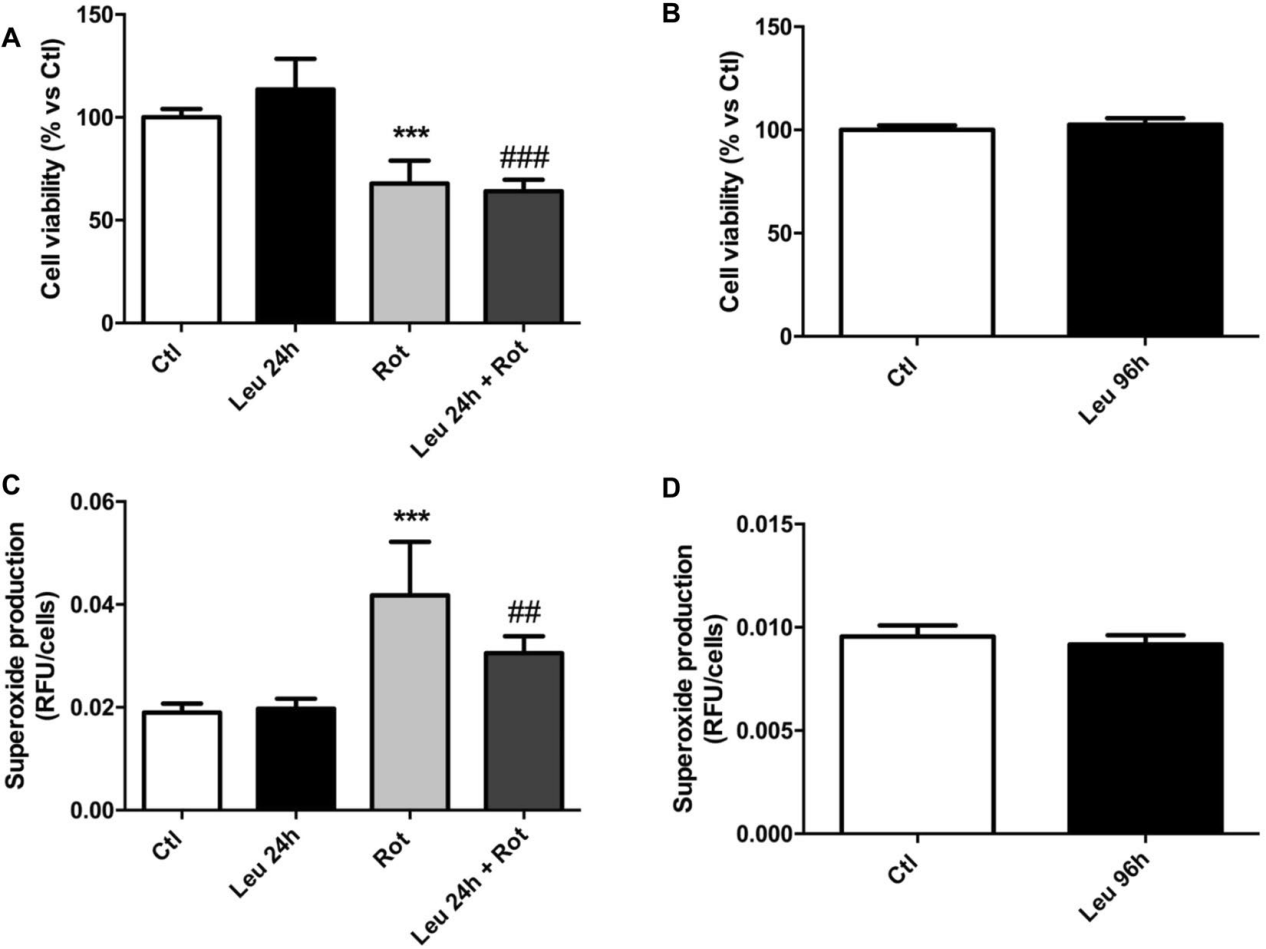
In vitro assay of Walker-256 cell viability and superoxide production in response to leucine treatment. Approximately 1 × 10^3^ W256 cells were seeded in 96-well plates and treated with 50 µM L-leucine for 24 (**A** and **C**) or 96 h (**B** and **D**). Cell viability was accessed using the neutral red uptake and the absorbance was normalised by the mean of a control group. Superoxide production was measured using dihydroethidium (DHE), and the fluorescence of each sample was normalised by the fluorescence of Hoechst 33342 (HO). Experiments were performed at least two times. Bar graphs represent the mean ± standard deviation. ***P < 0.001 *versus* Ctl; ^##^P < 0.01 *versus* Leu.

In the interest of elucidating the mechanism in which leucine induced oxidative stress and consequent cell death in tumour biopsies, we performed an *in vitro* study in order to evaluate cell death and oxidative stress (superoxide production) after treatment with leucine and whether leucine-treated cells would be more susceptible to an exogenous stressor, such as rotenone. However, different from tumour tissues, cells treated with leucine did not change their viability (Fig. 5). Moreover, cell death was increased (decreased viability) in both treated and non-treated cells after exposure to the exogenous stressor, rotenone (Fig. 5). Furthermore, superoxide production was similar in either leucine- or non-treated cells but was intensely enhanced when these cells were exposed to rotenone (Fig. 5).

## Discussion

In this study, we investigated the effects of the BCAA, leucine, on the Walker-256 carcinosarcoma metabolism. The main findings were a less aggressive tumour (lower FDG uptake and lactate production) in the presence of leucine and induction of a metabolic shift from glycolytic towards OXPHOS in both *in vivo* and *in vitro* models.

Most cancer cells present high rates of glucose uptake and aerobic glycolysis, a phenomenon known as the *Warburg effect*. This phenomenon is observed in the majority of cancer cells and is considered one of the hallmarks of cancer^9,10^. Even though it is inefficient regarding ATP production, aerobic glycolysis provides many advantages, such as a rapid and easily accessible source of glycolytic intermediaries that can be shunted into the pentose phosphate pathway and nucleotide synthesis, both of which are essential for cells with a higher proliferative rate. Moreover, this aerobic glycolysis causes an increase in lactate production and export, leading to acidification of the extracellular environment favouring metastasis formation^11^. In this study we found a decrease in ^18^F-fluorodeoxyglucose uptake and lactate exportation (lower expression of Ldha protein) in both the tumour biopsies and leucine-treated cells. Consequently, we found lower metastatic sites in tumour-bearing rats fed with a leucine-rich diet. This phenotype was followed by an increase in OXPHOS capacity of the tumours from the leucine-supplemented group.

This increase in OXPHOS capacity could be stimulated by acetyl-CoA, which is one of the final catabolic products of leucine metabolism. Since acetyl-CoA is directly consumed by mitochondria through the TCA^12^ cycle, leucine-treated cells favoured OXPHOS instead of glycolysis. The oxidative phenotype found in tumours biopsies from the leucine group was observed in both phosphorylating (ADP addition) and non-phosphorylating (CAT addition, mitochondrial uncoupling) states, which reflected an increase in oxidative capacity of these tumour biopsies. Accordingly, Walker-256 tumour biopsies and cells showed an increase in all bioenergetic parameters that were analysed in the functional respiratory assays.

In agreement with these results, leucine treatment caused an increase in the expression of mitochondrial genes (COX5a, CS, and ATP synthase) and proteins (CS and ATP synthase (Complex V) and UQCRC2 (Complex III)) associated with OXPHOS in the tumour biopsies. These molecular data agreed with higher mitochondrial density and area as observed by TEM images in the LW group.

Interestingly, leucine treatment induced metabolic reliance on OXPHOS also in Walker-256 cells, suggesting a direct effect of leucine on this tumour metabolism. Notably, our *in vitro* findings showed that leucine treatment boosts mitochondrial function as indicated by increased oxygen consumption rates and reduction in lactate levels. In line with these results, PGC-1α, NRF-1, COX5a, CS and CytC expressions, together with ATP synthase (Complex V) protein expression, were also significantly higher in leucine-treated cells, suggesting that leucine treatment also activated the mitochondrial oxidative phenotype *in vitro*.

Other studies have shown that leucine can increase OXPHOS and mitochondrial biogenesis in other types of cells, such as skeletal muscle, and change the metabolic profile in these cells^6,7^. The study performed by Vaughan and colleagues^8^ evaluated the effects of leucine treatment on oxidative and glycolytic metabolism in human and murine skeletal muscle cells. The authors observed a significant reduction in glycolytic metabolism and also on lactate export in leucine-treated cells. Therefore, Vaughan and colleagues concluded that leucine could potentially induce an oxidative profile by increasing the oxidative capacity in skeletal muscle cells. The blockage of lactate export may explain the alteration to a more oxidative phenotype^8^. In a previous study^13^, we found that lactate serum concentration was increased in Walker-256 tumour-bearing rats in comparison to healthy rats. This increase reflects high lactate production and its release by tumour tissues. Interestingly, under leucine treatment, tumour cells produced less lactate. Thus, as observed by Vaughan and colleagues^8^ in skeletal muscle cells, leucine also caused a decrease in Ldha expression, favouring an OXPHOS metabolism in tumours in both *in vivo* and *in vitro* experiments performed in this study.

It is also known that mitochondrial shape changes according to energy state^14^. In a glycolytic phenotype, the mitochondrial network appears interconnected, seems to be fragmented, or undergoes matrix expansion^15^. In fact, TEM images of Walker-256 tumour tissue showed a particular mitochondrial morphology, confirming to a glycolytic phenotype. On the other hand, the mitochondrial morphology of Walker-256 tumour tissue from leucine-treated rats was linked to an oxidative phenotype. The TEM images of mitochondrial morphology reinforce the hypothesis that leucine induces a metabolic shifting from glycolytic towards oxidative phosphorylation.

Besides metabolic shifting, we observed that a leucine-rich diet stimulates oxidative stress and apoptosis in Walker-256 tumour biopsies. A higher rate of OXPHOS produces not only ATP but also increases the oxidative stress^16^. We found higher MDA content in tumour biopsies from tumour-bearing rats fed with a leucine rich-diet. The increase in oxidative stress response was accompanied by a reduction in antioxidant response (decreased GST activity and higher MDA/GST ratio). Furthermore, a higher apoptosis index (a greater number of apoptotic nuclei) was observed in the LW group, in line with the increase in necrotic tumour mass observed by PET/CT images. Nakano and colleagues^17^ showed that BCAAs enhanced senescence in liver cancer, which was stimulated by DNA damage mediated through the mTORC1 pathway increasing the p21 translation, activated by p53^18^ leading to senescence and apoptosis. Accordingly, we observed an increase in protein expression of p53 in the tumour biopsies from the LW group. In fact, a study performed by Hagiwara and colleagues^19^ showed that BCAA supplementation decreased the incidence of liver cancer in both obese patients and diabetic model animals of carcinogenesis.

Considering that leucine led to an increase in apoptotic nuclei and a decrease in GST levels in tumour biopsies, isolated *in vitro* cells of Walker-256 tumours were used to elucidate this phenomenon. It was expected that cells treated with leucine would present higher cell death when exposed to exogenous oxidative stress, such as rotenone (mitochondrial complex inhibitor). However, cells treated with leucine (50 μM for 24 or 96 h) showed the same viability in comparison to non-treated cells and both groups had their viability diminished when the stressor agent rotenone was added. Thus, leucine treatment showed no effects on cell death or superoxide production within these *in vitro* experimental conditions. The fact that the results observed in tumour biopsies were not observed in isolated cells meant that probably other mechanisms underpinning this phenomenon could be acting differently from the *in vivo* study. Further studies should investigate the mechanism on how leucine could induce oxidative stress and apoptosis as seen in Walker-256 tumour tissue.

Other studies have evaluated the effect of leucine on cancer cell growth and metabolism. The study performed by Liu and colleagues^20^ evaluated the effects of leucine supplementation on pancreatic cancer growth in lean and overweight mice and found that leucine increased tumour growth. Xiao and colleagues^21^ assessed the effects of leucine deprivation in breast cancer in *in vitro* and *in vivo* models and showed that the deprivation of leucine inhibits and induces apoptosis in the selected breast cancer models. The different findings from these studies compared with the present one could be explained by some factors, such as different types of tumours and the presence or not of a cachexia state.

In conclusion, we describe for the first time the effects of a leucine-rich diet in Walker-256 tumour metabolism. Our data showed that leucine induced a metabolic shift from glycolytic towards OXPHOS in tumour biopsies and isolated cells. The metabolic shift was accompanied by enhanced mRNA and protein expressions of OXPHOS components, higher mitochondrial density, and area. Moreover, these metabolism alterations were associated with a decrease in tumour aggressiveness, suggesting that the leucine nutritional supplementation does not benefit this type of tumour. It is important to emphasize that the interpretation of our results considered only the Walker-256 tumour metabolism.

## Methods

### *In Vivo* experiments

#### Animals and diet

Adult female Wistar rats (approximately 90 ± 10 days old, obtained from the Animal Facilities at the State University of Campinas, UNICAMP, Brazil) were housed in collective cages under controlled environmental conditions (light and dark 12/12 hr; temperature 22 ± 2 °C, and humidity 50%–60%). The animals were monitored daily, weighed three times/week, and given food and water ad libitum. Semi-purified diets were made in accordance with the American Institute of Nutrition (AIN-93^22^) with the following components: The control diet (W) contained 18% protein and was composed of 20% casein (as a protein source), 39.7% corn starch, 13.2% dextrin, and 10% sugar (as carbohydrate sources), 7% soy oil (as a fat source), 5% cellulose micro fibre (as a fibre source), 3.5% salt mix, 1.0% vitamin mix, 0.3% cysteine, and 0.25% choline. The leucine-rich diet (LW) also contained 18% protein and was composed of the same amounts of casein, fat, fibre, salt, vitamin mix, cysteine, and choline as the control diet in addition to 3% leucine, 38.7% corn starch, 12.2% dextrin, and 9% sugar (as carbohydrate sources). The diets were normoproteic, isocaloric, and normolipidic.

#### Walker-256 tumour inoculation

The Walker-256 tumour is a well-established experimental model of cancer cachexia^23–26^. Walker-256 cells (2.5 × 10^6^ viable cells) were injected subcutaneously into the right flank of the rats. The general guidelines of the United Kingdom Co-ordinating Committee on Cancer Research, 1998 (UKCCCR)^27^ regarding animal welfare were followed, and the experimental protocol was approved by the Institutional Committee for Ethics in Animal Research (Comissão de Ética no Uso de Animais, Instituto de Biologia, Universidade de Campinas, Brazil -CEEA/IB/UNICAMP, protocol # 4289-1).

#### Experimental protocol

The animals were randomly distributed into two experimental groups (minimal of eight animals per group). The control group (W) was fed a control diet (18% protein), and the leucine group (LW) was fed a leucine-rich diet (18% protein + 3% leucine). At a pre-agonic moment (around twenty days following tumour inoculation), animals underwent 18F-FDG PET-CT imaging (four animals per group for this procedure). After 20 days of tumour evolution, the rats were euthanased. The tumour was removed and weighed. Some tumour fragments were immediately placed on ice-cold buffer containing 10 mM Ca-EGTA buffer for oxygen consumption measurements. Other tumour fragments were frozen directly in liquid nitrogen and stored at −80 °C for real-time polymerase chain reaction (PCR) and western blotting analyses, and additional fragments of tumour were immediately fixed in 2.5% glutaraldehyde and 2.5% paraformaldehyde in sodium cacodylate buffer (0.1M) at pH 7.4 and CaCl2 (3 mM) for 24 h on ice before being processed for electron microscopy analysis.

#### FDG-PET/CT imaging

The animals were fasting for 6 h prior to PET/CT scans. For a precise ^18^F-FDG injection and image acquisition, rats were anesthetised via intraperitoneal injection of ketamine (100 mg/kg body weight) and xylazine (12.5 mg/kg body weight)^18^.F-FDG (37MBq [1mC]) in approximately 0.5 mL of NaCl 0.9% solution was injected via the caudal vein. Radioactive activity was measured prior to the injection. Sixty minutes after ^18^F-FDG injection, each rat was subject to PET/CT imaging in the prone position. The scans were performed on the PET/CT imaging system (Siemens – Biograph mCT40). CT acquisition conditions were set to 70 kV, 155 mA, and 0.5-mm slice thickness. Each bed was scanned for 20 min from head to tail. A three-dimensional (3D) reconstruction model was used for analyses: OSEM 3D with 24 subsets and two interactions. For this, PET and CT images were fused through True D software (Siemens). Regions of interest (ROI) were drawn using the semi-quantitative method (Isocontour), this way we could determine the maximal ^18^F-FDG uptake (SUV_max_) in tumour areas and other tissues. SUV is defined as (A*W)/A_inj_, in which A (Bq/mL) is the radioactivity measured in a ROI, W (g) is the animal weight, and A_inj_ (Bq) is the activity of injected ^18^F-FDG. SUV_max_ is more accurate for estimating true SUV than SUV mean for this kind of analysis.

#### Walker-256 tissue sample preparation for oxygen consumption

The oxygen consumption was made according to the previous study performed by Busanello and colleagues^28^. Briefly, Walker-256 tissues were harvested from Wistar rats and placed on ice-cold buffer containing 10 mM Ca-ethylene glycol-bis (B-aminoethyl ether)-N′N′N′N′-tetraacetic acid (EGTA) buffer (2.77 mM of CaK_2_EGTA + 7.23 mM of K_2_EGTA, free concentration of calcium 0.1 mmol/L), 20 mmol/L imidazole, 50 mmol/L K^+^/4-morpholinoethanesulfonic acid, 0.5 mmol/L dithiothreitol, 7 mmol/L MgCl_2_, 5 mmol/L ATP, 15 mmol/L phosphocreatine, pH 7.1. Individual bundles from eight to eleven mg of tumour tissue were separated with forceps. Samples were permeabilised in ice-cold buffer containing saponin (50 μg/mL) for 30 min, gently stirred, and washed three times with MiR05 medium (60 mmol/L potassium lactobionate, 0.5 mmol/L EGTA, 3 mmol/L MgCl_2_, 20 mmol/L taurine, 10 mmol/L KH_2_PO_4_, 20 mmol/L HEPES, 110 mmol/L sucrose, 1g/L bovine serum albumin [BSA], pH 7.1) at 4 °C. Samples were dried with filter paper and weighed^29,30^.

Oxygen consumption was evaluated according to^29^ and^28^. Permeabilized tissues were added to a MiR05 medium containing EGTA (500 mM) at 37 °C supplemented with 10 mM glutamate plus 5 mM malate in an Oroboros oxygraph (Innsbruck, Austria). ADP (1 mM) and carboxyatractyloside (CAT, 12 μM) were added during the experiments.

#### Light and transmission electron microscopy

Tissue samples were removed from animals and immersed in a fixative solution (4% paraformaldehyde in 0.1 M phosphate-buffered saline [PBS]), pH 7.4, for 24 hours at 4 °C. Then, tissues were dehydrated in graded concentrations of alcohol, embedded in historesin (Leica Microsystems, Heidelberg, Germany) and sectioned at a width of 3 μm. The sections were mounted on slides and stained with haematoxylin and eosin. The sections were then examined for image analysis of apoptotic nuclei using a Nikon Eclipse E800 light microscope (Nikon Corporation, Tokyo, Japan). The apoptotic nuclei were counted using Image Pro-Plus Premium software (v.3.01, Media Cybernetics, Silver Spring, MD, USA) after capturing the image on a Leica microscope (Leica DMLM, Wetzlar, Germany) using 40× magnification. The number of apoptotic cells was determined by counting 20 fields on one slide from at least three samples per each group (W and LW).

For TEM, the tissue was immersed in a fixative solution consisting of 2.5% glutaraldehyde and 2.5% paraformaldehyde in a sodium cacodylate buffer (0.1M) at pH 7.4 and CaCl_2_ (3mM) for 24 h on ice. Tissue samples were then rinsed with cacodylate buffer/CaCl_2_ and were post-fixed in 1% OsO_4_ in sodium cacodylate buffer (0.1M), CaCl_2_ (3mM), and potassium ferrocyanide solution (0.8%) for 1 h on ice. Following that step, tissue samples were washed with milli-Q water and stained with uranyl acetate (2%) overnight at 4 °C. The next morning, tissue samples were washed in milli-Q water and dehydrated using an ethanol gradient. The samples were embedded in Epon 812 resin. Resin polymerisation was controlled in an incubator (60 °C) for 72 h. Ultra-thin sections were stained with uranyl acetate and lead citrate and then observed on a TEM LEO 906 (Zeiss), operated at 60 kV.

#### Quantitative RT-PCR

Total RNA from the Walker-256 tumour tissue and cells was extracted with TRIZOL^®^ reagent (Invitrogen) following the manufacturer’s instructions. The quality of the RNA samples was examined at 260/280 nm and 260/230 nm with a UV spectrophotometer (Nanovue Spectrophotometer 28923215 Ge BioSciences, USA). cDNA was produced using a high capacity cDNA reverse transcription kit (Applied Biosystems®, USA) containing Multiscribetm Reverse Transcriptase. cDNA synthesis was performed on 1 μg of RNA at 42 °C. Finally, cDNA was diluted 1:2 before its use in qPCR. Real-time reactions were performed using standard methods (ABI Prism 7500 Sequence Detection System; Applied Biosystems, Foster City, USA) and qPCR analysis was normalised to beta-actin. The genes evaluated using qPCR were PGC-1α (forward primer 5′-GACCACAAACGATGACCCTC-3′ and reverse primer 5′-TGTTGCGACTGCGGTTGT-3′), COX5A (forward primer 5′-TGTTGGCTATGATCTGGTTCC-3′ and reverse primer 5′-TTATGAGGTCCTGCTTTGTCC-3′), NRF-1 (forward primer 5′-TGCCCAAGTGAATTACTCTGC-3′ and reverse primer 5′-TCGTCTGGATGGTCATTTCAC-3′), CS (forward primer 5′-TATGGCATGACGGAGATGAA-3′ and reverse primer 5′-CATGGACTTGGGCCTTTCTA-3′), ATP5a (forward primer 5′-TGTTGCTTACCGCCAGATGT-3′ and reverse primer 5′-AGCAGGCGAGAGTGTAGGTA-3′), Cytc (forward primer 5′-AGGCAAGCATAAGACTGGAC-3′ and reverse primer 5′-ACTCCATCAGGGTATCCTCTC-3′) and succinate dehydrogenase (SDH) (forward primer 5′-ACCCCTTCTCTCTCTACCG-3′ and reverse primer 5′-AATGCCCGCTTCTCCTTGTAG-3′).

#### Western blotting

Samples of cultured tumour cells and tumour biopsies from Walker-256 tumour-bearing rats were lysed in RIPA buffer (150 mM NaCl, 25 mM Tris-Cl, pH 7,4, 0,1% SDS, 1% NP-40, 0,5% sodium deoxycholate) supplemented with protease inhibitor cocktail (Complete^®^, Roche), and protein concentration was measured by the Bradford method^3^. The proteins were separated by electrophoresis, transferred to nitrocellulose membranes, and the membranes were then incubated with primary antibodies against Ldha (CellSignaling, 1:1000), α-tubulin (Sigma-Aldrich, 1:5000), OXPHOS (Abcam 1:1000), Citrate Synthase (CellSignaling 1:1000), p53 (CellSignaling 1:1000), and vinculin (CellSignaling 1:1000) as a loading control. After that, the membranes were probed with secondary antibodies conjugated with peroxidase, and bands were visualised using a chemiluminescent reagent (ThermoFisher Scientific). The membrane images were captured using an image system (Amersham Imager 600, GE Healthcare), and band intensity quantitation was done using the Gel Pro software.

#### Tumour oxidative stress

Tumour tissue samples were weighed, homogenised in phosphate-buffered saline (PBS), and centrifuged for 15 min at 10,000 g. The supernatant was collected, maintained on ice, and assayed in duplicate. Tumour GST activity (nmol * min^−1^ * µg protein^−1^) was determined following the conjugation of 1-chloro-2,4-dinitrobenzene (CDNB) with glutathione^31^. The tumour lipid peroxidation product MDA was quantified using the substrate n-methyl-2-phenylindole (MPO)^32^. Once the GST activity and MDA content were determined, the MDA/GST ratio was calculated showing the intensity of oxidative process against the anti-oxidative response.

### *In Vitro* experiments

#### Glucose consumption and lactate production

For *in vitro* studies, cells from Walker-256 tumour-bearing animals were isolated from the intraperitoneal implant and maintained in culture. Briefly, the ascites fluid from a tumour intraperitoneal implant was collected, and the erythrocytes were lysed with 55 mM NH4Cl, 12 mM NaHCO3, and 0.1 mM EDTA, and the cell suspension was centrifuged at 500 × g, for 5 min, 4 °C. The supernatant was removed, and the pellet containing the tumour cells was seeded in 199 medium (Sigma-Aldrich) supplemented with 10% bovine calf serum (Lonza) and 1% penicillin/streptomycin (Lonza) and maintained at 37 °C and 95% O_2_–5% CO_2_ atmosphere with 85% relative humidity. Lactate production and glucose consumption *in vitro* analyses were done as a measurement of the lactate concentration released in the medium, and the glucose consumed was measured accordingly to the manufacturer’s instructions (Bioclin, Brazil). Briefly, cells were cultured in 12-well plates and treated with 50 µM L-leucine (Sigma, USA) for 24 h. The medium was removed and assayed for lactate production^1^ and glucose consumption^2^. Additional Walker-256 cells were seeded in 12-well plate and treated with 50 µM L-leucine (Sigma, USA) for 24 h for mitochondrial respiratory function analyses (Seahorse), protein extraction (western blotting), and RNA extraction (qPCR).

#### Mitochondrial respiratory function

The Seahorse analysis (XF24; Agilent Technologies Inc., Santa Clara, CA, USA) was performed according to the previous study performed by Lima and colleagues^33^. The oxygen consumption rate (OCR) was measured in accordance with the manufacturer’s instructions. Oligomycin (1 µM) was used to inhibit ATP synthase, and protonophore carbonyl cyanide m-chlorophenyl hydrazone (CCCP) (2 µM) was used in order to uncouple mitochondrial OXPHOS and rotenone (rot)/antimycin (AA) (1 µM) to block mitochondrial respiration and determine non-mitochondrial OCR. ATP-linked OCR was calculated by subtracting the uncoupled OCR (after the addition of oligomycin) from the basal OCR. Spare capacity was determined by subtracting basal OCR from the CCCP-induced OCR. Non-mitochondrial OCR values were subtracted from all data before being used for the analyses. All Seahorse measurements were normalised by protein quantified with respect to the Bradford assay.

#### Cell viability and superoxide production

Cell viability was accessed using neutral red uptake as as described by Repetto and colleagues^34^. Briefly, Walker-256 cells were seeded in 96 well plates at 1 × 10^3^ cells per well and allowed to attach overnight. On the next day, cells were treated with 50 µM L-leucine for 24 or 96 h, as indicated in the figure legends. Neutral red solution (40 µg × mL) was added and cells were incubated for additional 2 h. After that, the medium containing the staining solution was removed, and cells were washed twice with PBS. The stain was extracted from inside the cells by adding a solution of 50% ethanol, 49% H_2_O, and 1% glacial acetic acid. The absorbance was read at 540 nm.

Superoxide production was accessed using dihydroethidium (DHE, ThermoFisher Scientific). Walker-256 cells were seeded in 96 well plates at 1 × 10^3^ cells per well and allowed to attach overnight. On the next day, cells were treated with 50 µM L-leucine for 24 or 96 h, as indicated in the figure legends. DHE solution (10 µM) was added to the medium, and cells were incubated at 37 °C for 30 min, in the cell incubator. After that, Hoechst 33342 solution (1 µg × mL^−1^) (HO, ThermoFisher Scientific) was added, and cells were incubated for 15 min in the cell incubator. Cells were then washed twice with PBS, and DHE fluorescence was measured using an excitation/emission 518/605 nm; HO fluorescence was measured using an excitation/emission 350/461 nm.

### Statistical analyses

The statistical analyses were performed using the software Graph Pad Prism 6.0 (Graph-Pad Software, Inc). Data are expressed as mean ± standard deviation (s.d.) and analysed by t-test. P ≤ 0.05 was considered significant.

## Supplementary information

Supplementary Figure 1 and 2
