# Supplementary material for: Leucine-rich diet induces a shift in tumour metabolism from glycolytic towards oxidative phosphorylation, reducing glucose consumption and metastasis in Walker-256 tumour-bearing rats
Source: Sci Rep. 2019 Oct 29;9:15529. doi: 10.1038/s41598-019-52112-w (PMC6820796; doi:10.1038/s41598-019-52112-w)

Supplementary Figure 1 and 2

Manuscript title: Leucine-rich diet induces a shift in Walker-256 tumour metabolism from glycolytic towards oxidative phosphorylation, reducing glucose consumption and metastasis in Walker-256 tumour-bearing rats.

Authors: Laís Rosa Viana, Natália Tobar, Estela Natacha Brandt Busanello, Ana Carolina Marques, Andre Gustavo de Oliveira, Tanes I. Lima, Gabrielly Machado, Bianca Gazieri Castelucci, Celso Dario Ramos, Sérgio Q Brunetto, Leonardo Reis Silveira, Anibal Eugenio Vercesi, Sílvio Roberto Consonni and Maria Cristina Cintra Gomes-Marcondes

Supplementary Figure **1**. ***In vitro* study**: Walker 256 isolated cells treated or not with Leucine (50µM for 24 h). Related to Figure 2 in the main text

Crooped:

Ldha

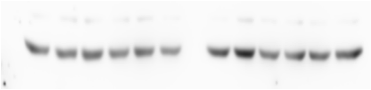

Tubulin

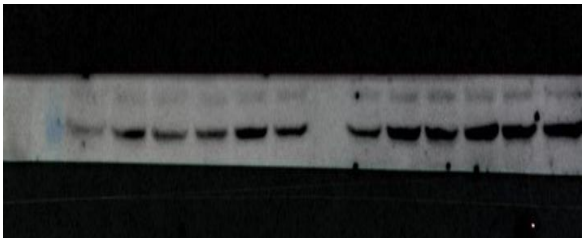

Control Leu

CV-ATP5A

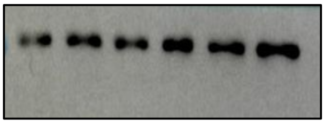

CIII-UQCRC

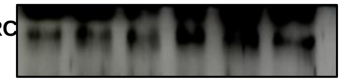

Vinculin

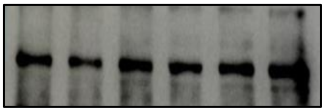

Original:

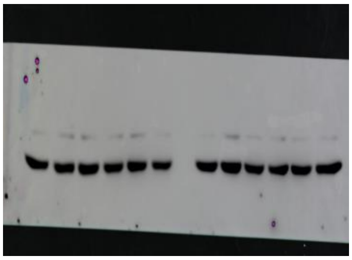

CV-ATP5A

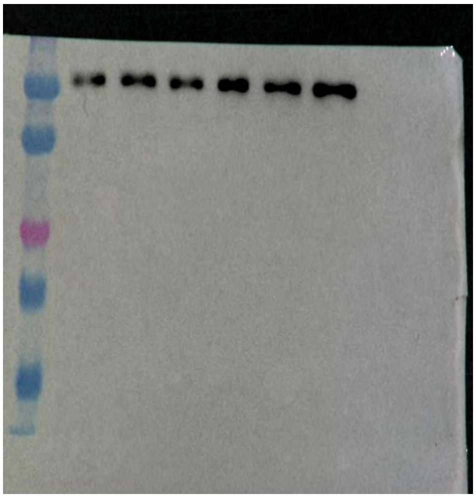

CIII-UQCRC2

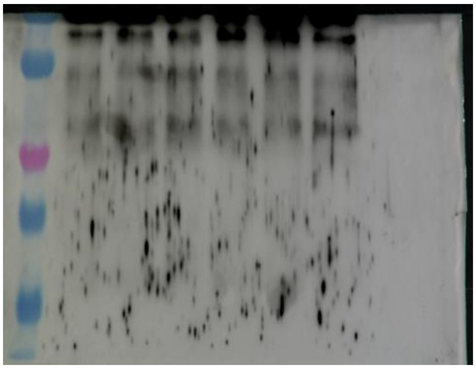

Vinculin

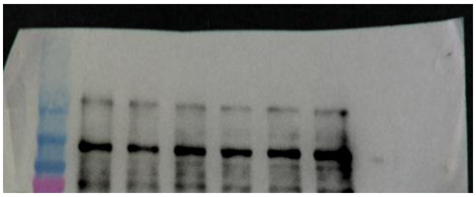

Supplementary Figure **2**. ***In vivo* study**:Protein expression in tumour biopsies from Walker-256 tumour-bearing rats . Related to Figure 3 in the main text

Crooped:

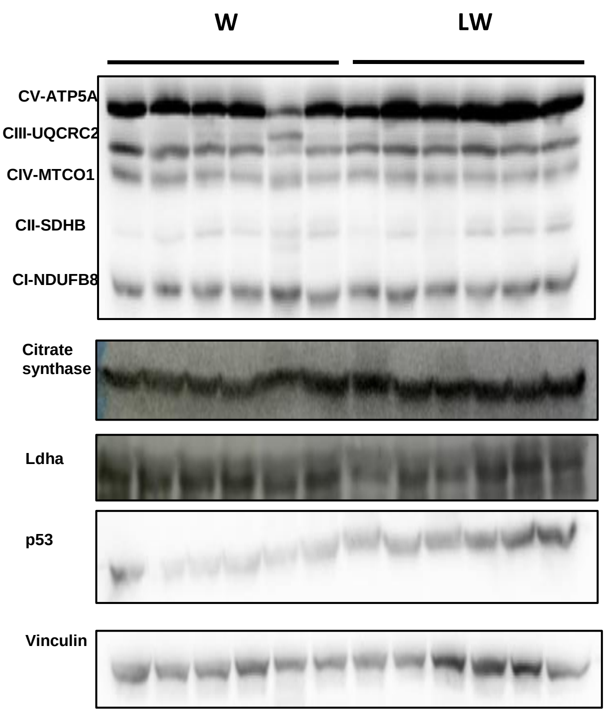

Original:

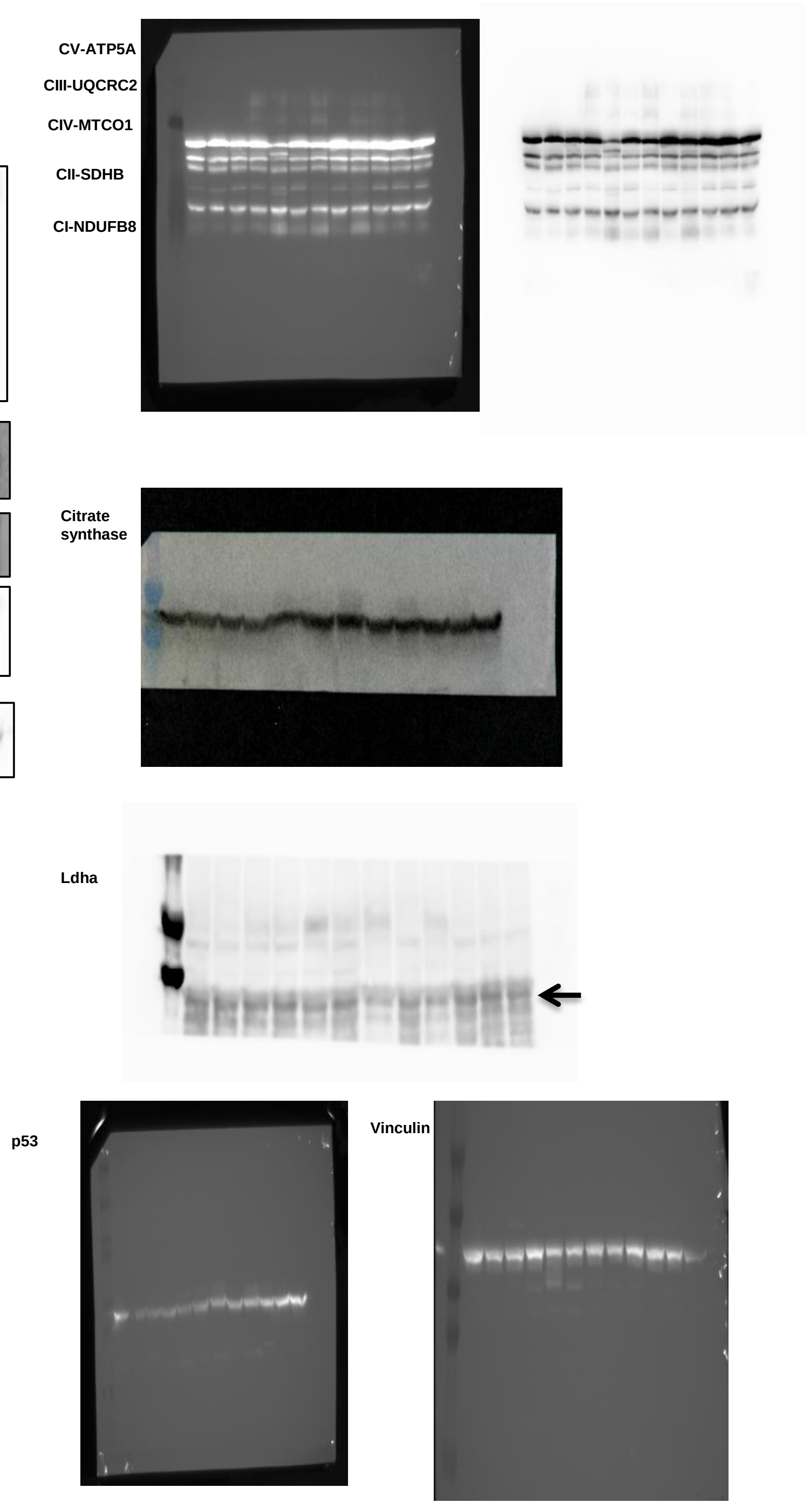

Supplement: Supplementary file 1 — Supplementary Figure 1 and 2 [file 41598_2019_52112_MOESM1_ESM.pdf]
